# Does medical school cause depression or do medical students already begin their studies depressed? A longitudinal study over the first semester about depression and influencing factors

**DOI:** 10.3205/zma001579

**Published:** 2022-08-04

**Authors:** Angelina Pelzer, Alexandra Sapalidis, Nadja Rabkow, Lilith Pukas, Nils Günther, Stefan Watzke

**Affiliations:** 1 University Hospital Martin-Luther-University Halle-Wittenberg, University Clinic and Polyclinic for Psychiatry, Psychotherapy and Psychosomatics, Halle/Saale, Germany

**Keywords:** depression, first semester medical students, BDI-II, mental health, risk factors, medical school

## Abstract

**Objective:**

In the past, medical students have been found to be at increased risk for depressive symptoms compared to the general population. This study addresses the question, whether medical students already show these elevated depression scores at the beginning of medical school or whether it is the studies of medicine that leads to symptoms of depression.

**Methods:**

In the winter semester 2018/2019, 148 medical students at a middle-sized German university answered a questionnaire during the first (T0) and last week (T1) of their first semester that examined various risk and resilience factors for initial depressive symptoms and their course. The severity of symptoms was assessed with the Beck´s Depression Inventory II (BDI-II). A subscale of the NEO-FFI was used to investigate the personality factor neuroticism.

**Results:**

Over the study period, the percentage of students suffering from at least mild depressive symptoms increased from 16.3% in the first week of their studies (T0) to 21.4% at the end of the first semester (T1). The use of drugs or medication, loneliness, mental overload, performance pressure and financial burden show the strongest influence on the development of depressive symptoms. Concerning surveyed resilience factors, in particular emotional support, physical workout and sufficient time for social contact appear to be protective. The more risk factors are concentrated on an individual, the higher its increase of depressive symptoms. The opposite is prevailing for the investigated resilience factors. Except for the use of drugs or medication, no other criterion than the BDI-II value at T0 was able to predict the BDI-II score at T1. This underlines that especially the interplay of factors is decisive.

**Conclusion:**

The findings of this study could indicate a worsening tendency of the BDI-II score in the further course of medical school. Ultimately, we emphasize the importance of offering preventive measures to medical students as early as possible.

## Zusammenfassung

**Zielsetzung:**

In vorangehenden Studien zeigte sich, dass Medizinstudierende im Vergleich zur allgemeinen Bevölkerung ein erhöhtes Risiko für depressive Symptome aufweisen. Diese Studie behandelt die Frage, ob Medizinstudierende bereits zu Beginn des Studiums erhöhte Depressionsraten aufweisen oder ob diese erst im Laufe des Studiums entstehen.

**Methoden:**

Im Wintersemester 2018/19 beantworteten 148 Medizinstudierende einer mittelgroßen, deutschen Universität einen Fragebogen während der ersten (T0) und letzten (T1) Woche ihres ersten Studiensemesters. Der Fragebogen untersuchte verschiedene Risiko- und Resilienzfaktoren für eine depressive Symptomatik und deren Verlauf. Der Schweregrad der Symptome wurde mit dem Becks-Depressions-Inventar II (BDI-II) erfasst. Eine Subskala des NEO-FFI wurde verwendet, um den Persönlichkeitsfaktor Neurotizismus zu erfassen.

**Ergebnisse:**

Während des Studienzeitraums nahm der Anteil an Studierenden, die unter mindestens milden depressiven Symptomen litten, von 16.3% in der ersten Woche des Studiums (T0) auf 21.4% in der letzten Woche des ersten Semesters (T1) zu. Der Gebrauch von Drogen oder Medikamenten, Einsamkeit, mentale Überforderung, Leistungsdruck und finanzielle Belastung zeigten den stärksten Einfluss auf die Entwicklung der depressiven Symptome. Was die untersuchten Resilienzfaktoren betrifft, so scheinen insbesondere emotionale Unterstützung, körperliches Training und ausreichend Zeit für soziale Kontakte protektiv zu wirken. Je mehr Risikofaktoren auf ein Individuum zutreffen, desto stärker nimmt die depressive Symptomatik zu. Bei den Resilienzfaktoren ist das Gegenteil der Fall. Abgesehen von der Einnahme von Drogen oder Medikamenten, sowie dem BDI-II-Wert zu T0, erwies sich kein weiterer Faktor als geeignet, den BDI-II-Wert zu T1 vorherzusagen. Dies weist darauf hin, dass der Interaktion der einzelnen Faktoren eine wichtige Rolle zukommt.

**Fazit:**

Die Ergebnisse dieser Studie weisen auf eine Verschlechterung des BDI-II-Wertes im weiteren Verlauf des Medizinstudiums hin. Letztlich möchten wir betonen, wie wichtig es ist, Medizinstudierenden so früh, wie möglich präventive Maßnahmen anzubieten.

## 1. Introduction

Over 300 million people worldwide currently suffer from depression [1]. In Germany, every tenth adult is affected by recent depressive symptoms [2]. These are also particularly common in medical students and have also been associated with medical malpractice in interns, such as preventable medication errors [3], [4] so timely recognition of symptoms is especially important.

Because there is sparse evidence on whether depression first occurs during medical school or is already present in novice students, this study aims to contribute to a better understanding of the factors that are associated with depressive symptoms in first-year medical students.

Studies showed that medical students represent a special risk group for depression with an average prevalence of 27.2% of at least moderate depressive symptoms [3], [5] which is significantly higher than the prevalence in the general population [6]. Here, age groups between 18 and 29 years, which also include the considerable majority of the medical students, only had a prevalence of 16.4% in women and 9.5% in men [2]. Solely female gender increases the lifetime risk of depression [7], but ultimately it is a result of an interaction of biological factors and environmental exposures [8]. In the vulnerability-stress model, vulnerability describes the multifactorial mediated disposition to depression, while stress refers to life events [9]. Such a stressful life event may also be the start of university education, since students are exposed to new burdens at this point of time. Previous studies from our group found elevated levels in first-year students as early as the end of the first semester [10]. Therefore, it is necessary to take a closer look at the people, who are starting medical studies. Admission in Germany is primarily based on the grade of the general university entrance qualification. To a lesser extent, professional medical training, the results of a medical aptitude test (TMS) and the waiting period for a study place are also taken into account [11]. Students selected by those methods were found to have a different susceptibility to depression depending on their personality structure [12]. For example, people with a combination of traits of a high level of neuroticism and a high degree of conscientiousness in medical school are particularly vulnerable to stress [13]. Other predisposing factors include a negative self-perception, low self-esteem, a lack of optimism and an insecure attachment style [14], [15].

Due to a so far ambiguous data situation, the following question arises for us: Do medical students show increased depressive symptoms already at the beginning of their studies or is the development of such symptoms linked to certain influences from the course of studies? 

Our aim is to contribute to a better understanding of the reasons for the high prevalence of depression in first-year medical students. Furthermore, we want to investigate in more detail at which point in time symptoms of different severity are already present and which factors have a triggering effect.

## 2. Methods

### 2.1. Study design and setting 

To identify potential changes in the severity of depressive symptoms within the first semester, we conducted a longitudinal study during the first and last week of the semester in a cohort of first-year medical students. In order to cover the first-year students as completely as possible, the questionnaires were distributed in the compulsory courses. Respondents were informed in advance about the aims of the study, and there were no incentives for the voluntary and anonymous study participation. The questionnaire was preceded by an information sheet about the scope of the study and existing support services (see attachment 1 and attachment 2 ). Completing the questionnaire took 30 minutes and was done in privacy at the end of the respective class. Alternatively, participants could complete the questionnaires outside of class and then drop them into prepared boxes on campus. The aim was to enable anonymous return of the filled or blank questionnaires, in case of refusal to participate in the study. The regulations on confidentiality and data protection were also warranted by using an anonymous code for the study participants, which made it possible to individually link the data collected first with those of the second assessment at the end of the first semester. For this, students generated a pseudonym according to a given scheme. Ethical approval for the survey questions and methods was obtained from the local ethics committee (approval ID 2017-138) prior to the start in the winter semester 2018/2019. 

#### 2.2. Participants

Among the 236 students who started studying medicine in the winter semester 2018/2019, there were no exclusion criteria. A number of 220 students participated in the first assessment (T0) within the first week of the study, representing 93.2% of the target population. However, only 148 medical students took part in the second assessment (T1) at the end of the semester (62.7%). This was due to mandatory regulations of attendance, that allowed students to miss one of the courses or due to rejection of participation at this stage of the study. Data from students who participated only in the first survey were excluded.

N=156 subjects (70.9%) of the initial sample were biologically female and N=64 participants (29.1%) were biologically male. This proportion does not differ from the distribution within the population of all 236 students who started studying medicine at the Martin-Luther-University in the winter semester 2018/2019 (χ^2^[df=1] =.349; p=.555). Comparatively, the German wide average for female medical students in the first semester of 2018 was 62.0% [14]. In three cases, sex did not match gender. Age ranged from 17 to 36 years with a mean of 20.5 years (SD=3.43). The remaining n=148 subjects at T1 did not differ from the T0 sample in the distribution of subjects in either sex (χ^2^[df=1] =1.404; p=.236) nor age (F[df=1] =.670; p=.414).

#### 2.3. Instruments

##### 2.3.1. Trait related risk factors 

Sociodemographic data such as age, sex, gender, relationship status, origin, family status and previous vocational training were assessed in a questionnaire. Hereafter, potential risk factors were examined, including parents' education as well as occupation and thus their socio-economic status. The latter was classified as low, and hence as a hazard factor, if the careers of both parents were described as “without a degree”, “secondary school leaving certificate” or “unqualified”. It was also asked about the loss of a parent through death or divorce. Moreover, the personal history of mental illness was examined. A family predisposition was assumed, if at least one first- or second-degree family member was receiving treatment for a psychological disorder other than dementia. 

To assess the personality trait neuroticism, we used the standardized neuroticism subscale of the NEO-FFI [16] as a self-assessment instrument. This trait is associated with nervousness, anxiety, susceptibility to stress and irritability, therefore a high expression can be considered a risk factor for depressive disorders [17]. The German version [18] of the questionnaire consists of 12 statements, each statement is evaluated with the help of a Likert-Scale. Thereby the possibilities of the evaluation range from strong rejection (0/4) to strong agreement (4/4). The application of this screening tool provides an objective, reliable and well validated result [19].

##### 2.3.2. Stress factors 

In the following presentation of the results, a theoretical distinction is made between general stress factors and study-related stress factors. The latter include a workload that is subjectively assessed as high, little flexibility in the organization of studies, pressure to perform, lack of time, excessive demands, loneliness, competition among students, and an uncertain future.

Financial burden, as one of the following general stress factors, was defined by at least “sometimes having too few financial resources”. Besides, data on substance abuse of all kinds was collected. In accordance with Hodgson et al. [20], alcohol intake was considered risky if a woman consumed more than six alcoholic beverages (with one drink equivalent to 250ml of beer, one glass of wine, or 2cl of liquor) per occasion more than once a month or a man used more than eight. Additionally, the use of drugs or medication to improve concentration, sleep or sedation was asked.

##### 2.3.3. Resilience factors

Protective factors were elicited with questions about motivation to enroll and attitudes toward the decision to pursue this course of study [21]. The participants’ existing social network was analyzed with regard to relationship problems, sufficient social contact despite physical distance to friends or family and emotional support [22]. Apart from that, the hours per week spent on hobbies, physical exercise [23] and playing a musical instrument [24] were determined. Also, it was asked about regular meals and healthy nutrition [25], as well as one’s own body perception. Finally, the application of specific mental or physical relaxation techniques such as yoga or autogenic training has been explored [26].

##### 2.3.4. Beck-Depression-Inventory II (BDI-II) 

Finally, we used this self-report instrument to assess the severity of depressive symptoms [27]. The testing procedure can be used in both clinical and non-clinical settings and is considered objective, reliable, and valid [28]. The severity of 21 symptoms is rated on a four-point scale, where 0 meaning “does not occur at all” and 4 meaning “occurs at the highest intensity”. The total BDI-II-score was used as the primary outcome of this study. This total-score can be classified as “no depression” (0-8 points), “minimal depression” (9-13 points), “mild depression” (14-19 points), “moderate depression” (20-28 points) or “severe depression” (29-63 points). Several previous studies provide comparative values, such as a survey of 12677 college students, in which the mean total score was 6.99 (SD=7.56) [29] or another group of 15233 students with a mean BDI-II score of 9.14 (±8.45) [30].

#### 2.4. Statistic procedures

Data were examined using IBM SPSS 25.0. Descriptive data are presented with mean and standard deviation or relative frequency. Differences between the T0- and T1-samples in terms of nominal data were tested with the Chi^2^-statistics. For metric data, analyses of avaice were used. 

BDI-II scores were analyzed for normal distribution using the Kolmogorov-Smirnov-Test. Since both scores at T0 and T1 were positively skewed (both p<.001), non-parametric statistics such as the Wilcoxon signed rank test for connected data and the Spearman rank correlation were used to test for repeated measures and correlation between risk or protective factors and depression. In the case of T1 assessment, partial correlation with BDI-II total score at T0 was controlled. Prediction of BDI-II scores was estimated using stepwise linear regression models (p_in_=.05; p_out_=.10). For prediction of BDI-II total score at T1, initial BDI-II total score at T0 was included at a first step, respectively. To test whether prevalence of stress factors changed between T0 and T1, McNemar tests for paired nominal data were computed [31]. 

Missing data occurred in “familiy burden with mental disorder” and “grew up outside Germany” (each n=13) as well in “use of drugs or medication” (n=2). Missings were not imputed.

## 3. Results

The mean result of BDI-II score at T0 was M=8.28 (SD=6.69). 82.7% showed no or minimal symptoms, 9.6% had mild symptom scores and 7.7% showed moderate or high scores.

At T1 BDI-II-total was at mean M=10.12 (SD=8.44). 78.6% showed no or minimal symptoms, 6.9% had mild symptom scores and 14.5% showed moderate or high scores.

This difference was statistically significant (Wilcoxon Z=-1.96; p=.050). Spearman rank correlation between T0 and T1 BDI-II-total scores was r=.65 (p<.001). Change of BDI-II ranged from -12 points to +23points with a mean of +1.23 (SD=6.10).

Students reported a mean of 2.04 risk factors (SD=.98) at T0 (cf. table 1 (Tab. 1)). Individual sum of risk factors correlated with BDI-II total at T0 with r=.37 (p<.001). Partial correlation (corrected for BDI-II total score at T0) between sum of risk factors and BDI-II total score at T1 was r_p_=.19 (p=.038). Linear regression (stepwise p_in_=.05, p_out_=.10) to predict BDI-II total score at T0 showed that neuroticism, female sex and family burden explained 44% of variance. For prediction of BDI-II total score at T1, a linear regression model with BDI-II total score at T0 (inclusion) and the risk factors (stepwise) was calculated. However, besides BDI-II total score at T0 (R^2^=.50), no additional predictor was significant. 

Students reported a mean value of M=1.96 (SD=1.31) stress factors at T0 (cf. table 2 (Tab. 2)). The sum of individual stress factors at T0 correlated with the BDI-II total score at T0 with r=.45 (p<.001), the partial correlation with the BDI-II total score at T1 (corrected for BDI at 0) was r_p_=.21 (p=.016).

Linear stepwise regression to predict the BDI-II total score at T0 yielded that the use of drugs or medication, loneliness, mental overload, performance pressure and financial burden predicted the criterium and together explained 32% of variance.

At T1, a mean of M=2.35 stress factors (SD=1.44; Wilcoxon Z=-3.33; p=.001) was reported by students. Sum of stress factors at T1 correlated with BDI-II total score at T1 with r=.47 (p<.001).

The following factors changed over time (McNemar test for nominal data): time pressure (p=.001), performance pressure (p<.001), alcohol consumption (p<.001) and in tendency use of drugs or medication (p=.070). 

For prediction of BDI-II total score at T1, again a linear regression model with BDI-II total score at T0 (inclusion) and the stress factors at T0 (stepwise) was applied. Additionally, to BDI-II total score at T0 the use of drugs or meds showed to be a significant predictor with an incremental explanation of variance of R^2^=.10. 

Students reported M=3.67 (SD=1.36) resilience factors at T0 (cf. table 3 (Tab. 3)). Sum of individual resilience factors at T0 correlated with BDI-II total score at T0 with r=-.37 (p<.001), partial correlation with BDI-II total score at T1 (corrected for BDI at T0) was r_p_=-.10 (not significant).

Linear stepwise regression to predict BDI-II total score at T0 showed that emotional support, physical exercise and sufficient time for social contact predicted the criterium and explained a variance of 30%.

At T1, students reported M=3.00 resilience factors (SD=1.46). Change over time was significant (Wilcoxon Z=-5.70; p<.001). Sum of resilience factors at T1 correlated with BDI-II total score at T1 with r=-.48 (p<.001).

McNemar tests showed significant changes between T0 and T1 for satisfaction with choice of studies (p<.001), active playing of music instrument (p=.001), and physical exercise (p<.001). 

However, besides BDI-II total score at T0 (R^2^=.50), none of the resilience factors at T0 explained additional variance of BDI-II total score at T1.

## 4. Discussion

The aim of this research was to investigate whether medical students already show depressive symptoms at the beginning of their studies through predisposing factors or whether factors correlated with the BDI-II score are more due to the course of study. 

Our measured BDI-II scores significantly differed between first week and end of the first semester. Overall, we found that the BDI-II mean score was higher at the end of the first semester compared to the beginning of medical school, with scores at T0 and T1 positively correlated with each other. 

Although both mean values are in the range of no or minimal depressive symptoms, the change over a period of only five months indicates a worsening trend, as has already been demonstrated in several studies [32], [33], [34].

Moreover, already 16.3% of students, initially showing at least mild depressive symptoms, is a higher percentage than expected in comparable age (18-29 years) and educational stratum of the general population in Germany [2]. Although this comparison must consider the different gender distribution in our study collective, studies in other countries across continents also indicate an increased prevalence of depression in medical students [35], [36], [37].

It should also be noted that the percentage of students with moderate or high scores (BDI-II>19) almost doubled in the second investigation. Since the proportion of students with no or minimal symptoms remained about the same, but the group with mild symptoms became notably smaller, an increasing deterioration can be assumed, especially in the latter mentioned group. This suggests that students who exhibit depressive symptoms early in their studies might benefit from receiving psychological support at an early stage, as exacerbation could be prevented [38], [39], [40]. 

While the sum of risk factors also correlates with the BDI-II score at T0, neuroticism, female gender and family burden, had the greatest influence, consistent with the results of previous studies [13]. Female medical students are more likely to suffer from depression compared to their male peers [7], which is worth highlighting given the gender imbalance of 70.9% females in our study population.

Our findings on the influence of family burden could represent psychosocial, genetic as well as socioeconomic components in the pathogenesis of depression [41], [42], early special education for students at risk, could therefore be discussed [43].

Growing up abroad correlated positively with the BDI-II score at T0 (cf. table 1 (Tab. 1)). It is assumed that additional stresses, such as structural racism, social isolation due to separation from family, or a language barrier, have an impact on mental health in foreign students [22], [44], [45]. However, since only 7.3% of our surveyed students have a foreign background, any interpretation from this data is very limited. Whereas growing up in eastern federal states correlated negatively with the BDI-II value at T0 and concerned 67.6% of students. As the surveyed university is also located in eastern Germany, we assume that location dependent circumstances like an already existing social network initially appear protective [22].

In the end no risk factor alone, but only the BDI-II value at T0, allowed to make an assumption about the expected BDI-II value at T1.

With regard to the investigated study-related stress factors loneliness, mental overload and performance pressure had the biggest association with the BDI-II score at T0. In particular, the number of students suffering from time pressure and the pressure to perform increased over time to T1 (cf. table 2 (Tab. 2)). Concerning this change, the examination phase at the end of the semester additionally has to be taken into account. 

Among general stress factors, the use of drugs or medication and financial burden showed the strongest influence on the BDI-II score. Even though semester fees in Germany are low in international comparison, some students are affected by a financial burden, whose association with the occurrence of depressive symptoms is already known from studies in other countries [36], [37]. The use of drugs or medication even proved to be an additional significant predictor of BDI-II score at T1. Although the prevalence of 10,1% at T1 is still rather low, it is striking that the percentage of drug or medication abuse in our sample more than doubled compared to T0. 

Between the use of drugs or medication and depression, a mutual relationship can be found. On the one hand, consumption is often viewed as a kind of self-medication for a depressive state. On the other hand, chronic or early drug abuse can lead to neurobiological changes that increase the risk of depression [46]. Substance abuse to improve concentration, sleep or sedation can be seen as an attempt to be even more efficient in order to meet the high academic requirements of medical school [47]. However, a more precise differentiation between prescribed medication or illicit drug use is needed in future research.

We explain the lack of a positive correlation between the consumption of the other surveyed drug, alcohol, at T0 and the BDI-II score at T0 by suggesting that alcohol use in this case may reflect rather social integration [48]. 

As expected, the reported resilience factors correlated negatively with the BDI-II score at T0. The only exception is the use of relaxation techniques, such as yoga or meditation, which shows a direct correlation with the measured BDI-II values. This contradicts previous findings that such relaxation techniques can reduce stress and anxiety [26], [39]. However, since the sample of students applying relaxation techniques is very small, and the frequency of use was not asked, the interpretation of this result is very limited. Nevertheless, it could be assumed that especially students who were affected by stress or depressive symptoms used these techniques, as they were presented and taught in previous university courses.

 Instead, emotional support, physical exercise and sufficient time for social contacts in particular show a negative association with depressive symptoms. The protective effect of social support [49] and physical activity [23] has already been shown. But in the further course, the number of students participating in sports decreased, as well as the number actively playing a musical instrument, with lack of time likely a major cause [50]. This leads to the conclusion that a restructuring of the organization of medical school should be considered in order to create the necessary space for leisure time and sporting activities in the future. 

Furthermore, especially satisfaction with the choice of study decreased, with the reasons for which the decision for a medical career was made playing a role. For instance, students, who chose to study medicine because of a potentially over average income or prestige in society, developed higher depression scores than students who made this decision primarily because of a basic interest in medicine itself [21].

Finally, it can be summarized, that the more risk or stress factors are present, the higher the measured BDI-II value. The opposite is the case with resilience factors, which lower the BDI-II value.

## 5. Limitations

It has to be pointed out that the study interval is rather short at five months, which is equivalent to one semester. Consequently, the BDI-II scores are highly correlated, as the time interval allows little change. However, the focus on this period was deliberate because there are already several studies that have examined the entire course of medical school. Another concern is, if T0 is not already too late to answer the question of whether medical students already show depressive symptoms before the start of their studies. Although it can be assumed that there is less academic pressure to perform in the very first week of studies, many other factors, such as moving to an unfamiliar environment, must already be taken into account. A more precise answer would have been provided by an initial data collection during e.g., already high school. Unfortunately, this is difficult to implement in reality, as only very few students can have the certainty of a study place so far in advance. With regard to T1, it should be noted that it took place during an exam period. Thus, it cannot be excluded that the deterioration of the BDI-II values is caused by higher a higher stress level at this time and recedes as soon as the phase is over. To avoid this influence in a similar survey in future, the beginning of the second semester could also be considered as a second measurement point. A further limitation is the decreased sample size from 220 at T0, which almost completely represents the cohort of first-year medical students, to 148 at T1 (60.4%). Consequently, since selection or volunteer bias among medical students cannot be ruled out, the generalizability of the results is limited. Hence, further development of the test strategy is required in order to achieve a higher response rate in the follow-up survey. It is also noticeable, that the study took place at one single university in Germany. As a recent survey shows, depressive symptoms occur significantly more frequently in German young adults than in the European average [51]. The transferability of the data to medical students in other universities or countries is therefore questionable.

Furthermore, the variables included should be taken into account. Although many influencing factors were investigated, it is hardly possible to consider their entire diversity.

For example, a differentiation between illicit drug use and prescribed medication would have been more precise. As our study demonstrates a big impact of substance consumption on the BDI-II score, further research should specify in this topic. 

Finally, when collecting data with a questionnaire, various biases occur due to social desirability [52] or an increase in the correlation of variables when they are collected together [53]. 

Concerning the BDI-II as a measuring instrument, it must be pointed out that the use of a self-report questionnaire cannot replace a professional diagnosis of depressive illness. Nevertheless, there is high agreement between the clinical diagnosis of depression and the assessment by the BDI-II [28], [54]. 

## 6. Conclusions

Within this study, 16.3% of students already experienced at least mild depressive symptoms during the first week of medical school. By the end of the first semester, this proportion had increased to 21.4%. With respect to this rise in depressive symptoms at the end of the first semester, the present study can only identify causes to a limited extent. It is important to note that the central predictor of depressive symptoms at T1 is depressiveness at T0. This highlights the need to pay attention to, recognize, and address depressiveness at baseline, as especially the begin of studies seems to be a vulnerable time. Educational and preventive offers therefore should be provided from the very beginning. Factors such as loneliness, pressure to perform, competitive pressure, financial stress, excessive demands, and substance abuse are associated with a worsening of depressiveness. These observations and moreover the significantly higher incidence of medical malpractice among depressed physicians underscore the importance of universities already creating structures that enable future physicians work in a healthy manner [55]. 

However, no factor besides drug use contributes separately to the development of depressive symptoms. This can be interpreted as meaning that especially already high initial depressiveness increases the risk of being lonely in the course of the study, due to social withdrawal. It also raises the likelihood of feeling under pressure to perform and being overwhelmed, leading to negative self-assessment and evaluation of the future. Ultimately, more competitive pressure is felt as well, due to a negative evaluation of the environment. These observations are consistent with Beck's cognitive triad and are subsequently associated with worsening depressive symptoms [56]. Thus, there is not a single particular determining risk or resilience factor that defines the development of depression among medical students. Although a certain selection of specific personality traits could promote depression, these alone would usually not lead to an illness. However, risk constellations can be found, that make the occurrence of depressive symptoms significantly more likely [57]. In the same way, medical studies can be regarded as psychologically very stressful, but can be mastered well with the help of a healthy coping style [49]. However, if the individual's personal resources are not sufficient to withstand the great pressure, this can result in the development of a depressive illness. Therefore, future research should focus on developing methods to identify students who are particularly at risk as early as possible and provide them with effective support services.

## Abbreviations

- TMS: Test for medical courses of study
- e.g.: exempli gratia
- BDI-II: Beck's Depression Inventory II
- NEO-FFI: Neuroticism, Extraversion, Openness - Five Factor Inventory
- CI: Confidence Interval
- Cf.: Confer
- SD: Standard Deviation
- ANOVA: Analysis of variance

## Author contributions

The authors contributed to the organization and implementation of the study questionnaires.

## Declarations

### Ethics approval

The survey was performed in accordance with the Declaration of Helsinki. Ethics approval was obtained from the ethics committee of the Martin-Luther-University Halle-Wittenberg in 2018.

#### Informed consent

Informed consent has been obtained from all participants before study-related activities began.

#### Availability of data and materials

The datasets used or analysed during the current study are available from the corresponding author on reasonable request.

## Acknowledgement

We want to express our gratitude to the medical students who participated in this survey.

## Competing interests

The authors declare that they have no competing interests. 

## Supplementary Material

Sociodemography, Risk & Resilience. Self-description questionnaire (T0)

Sociodemography, Risk & Resilience. Self-description questionnaire (T1)

## Figures and Tables

**Table 1:**
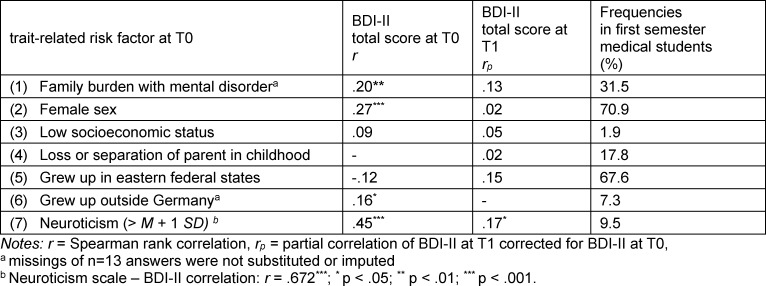
Pearson correlations between trait-related risk factors at T0 and BDI-II total score, prevalence of trait-related risk factors in the study sample

**Table 2:**
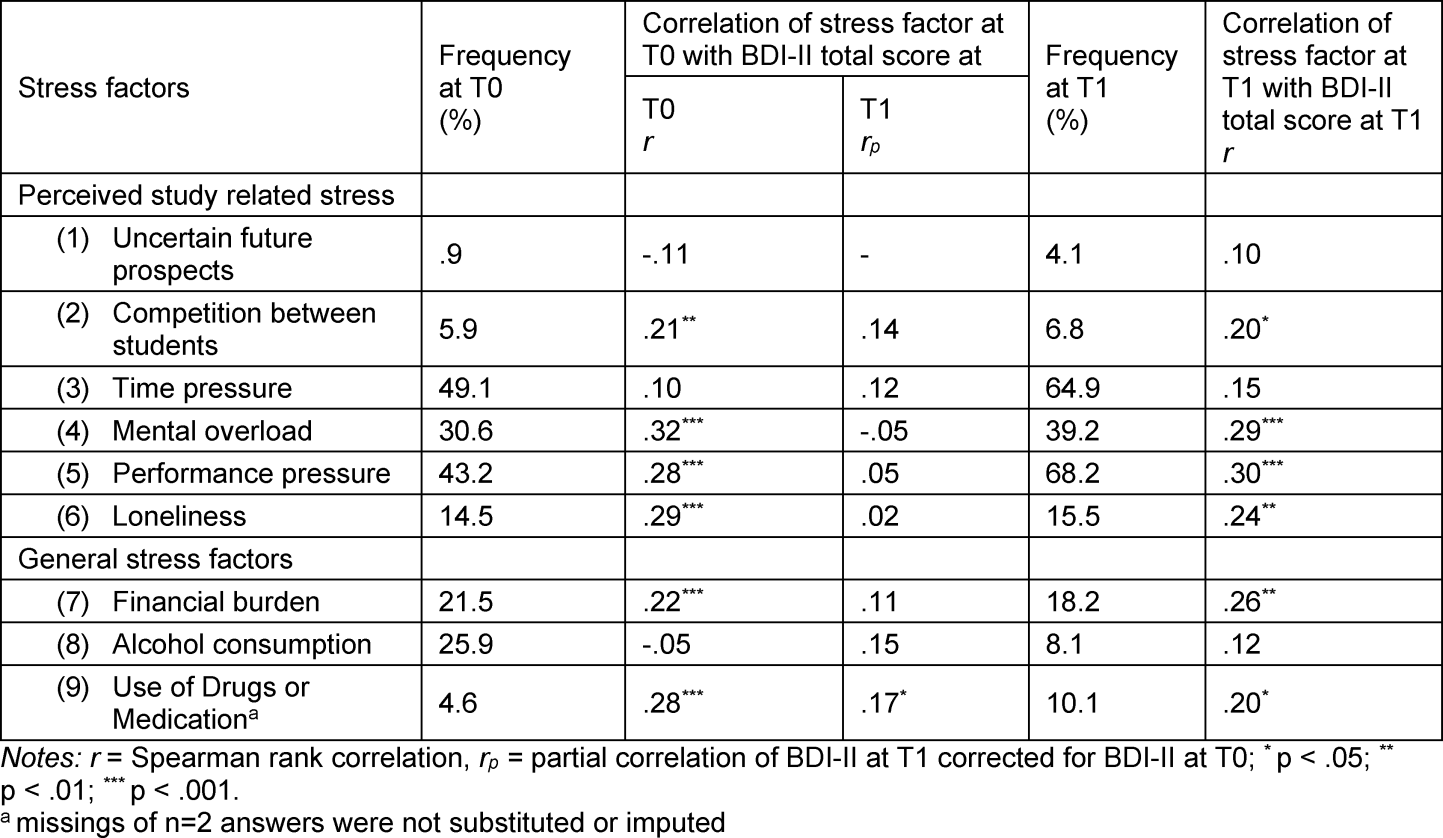
Pearson correlations between stress factors at T0 and BDI-II total score, prevalence of stress factors in the study sample

**Table 3:**
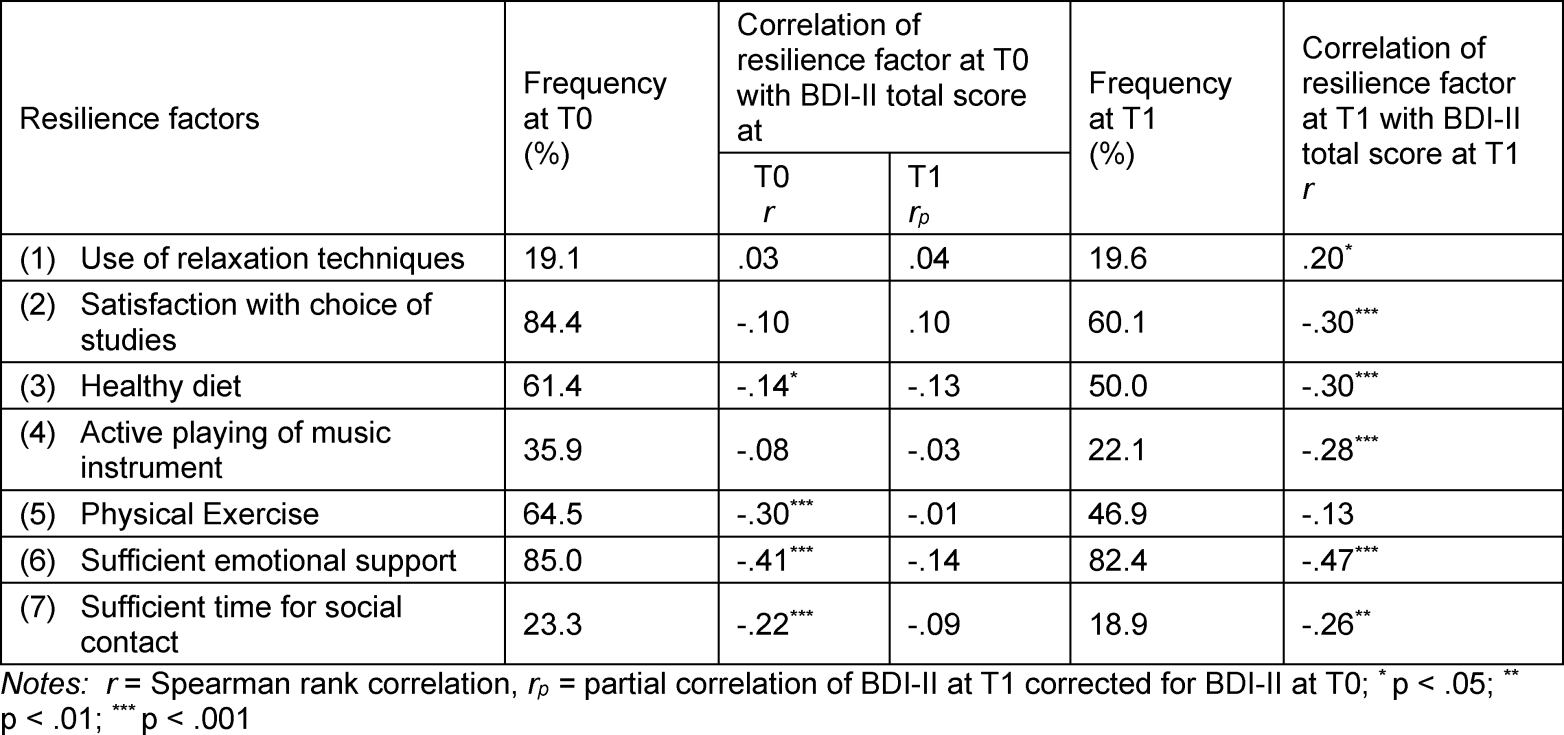
Pearson correlations between resilience factors at T0 and BDI-II total score, prevalence of resilience factors in the study sample
