# Supplementary material for: Does medical school cause depression or do medical students already begin their studies depressed? A longitudinal study over the first semester about depression and influencing factors
Source: GMS J Med Educ. 2022 Aug 4;39(5):Doc58. doi: 10.3205/zma001579 (PMC9733480; doi:10.3205/zma001579)
Supplement: Sociodemography, Risk & Resilience. Self-description questionnaire (T0) [file JME-39-58-s-001.pdf]

Self-description questionnaire T0

SOCIODEMOGRAPHIC QUESTIONS
STRESS & RESILIENCE FACTORS

PNUM: (assigned by the study team) Date:

Please answer each of the following points by ticking the appropriate alternative or by entering the appropriate value

1. Biological gender
- Female ☐
- masculine ☐

2. Social gender
- Female ☐
- masculine ☐
- both ☐
- neither ☐
- different ______

3. age in years ☐

4. size in cm ☐

5. weight in kg ☐

6. civil status
- single ☐
- married ☐
- separated or divorced ☐
- widowed ☐

7. Relationship regardless of marital status
- no partner or relationship shorter than 3 months / living without a partner ☐
- married and living together ☐
- not married but living together ☐
- Partnership without living together (> 3 months) ☐

8. Duration of the relationship in months (0 if no partnership) ☐

9. Number of biological children ☐

10. In which state did you grow up?
- Baden-Wuerttemberg ☐
- Bavaria ☐
- Berlin ☐
- Brandenburg ☐
- Bremen ☐
- Hamburg ☐
- Hesse ☐
- Mecklenburg-Western Pomerania ☐
- Lower Saxony ☐
- North Rhine-Westphalia ☐
- Rhineland-Palatinate ☐
- Saarland ☐
- Saxony ☐
- Saxony-Anhalt ☐
- Schleswig-Holstein ☐
- Thuringia ☐

11. What is your mother's highest school-leaving qualification?
- Without school graduation ☐
- secondary schools ☐
- Realschule (10th grade) ☐
- High School ☐

12. What is your mother's highest professional qualification?
- unskilled ☐
- Vocational training ☐
- Technical / university studies ☐

13. What kind of work is your mother currently doing?
- Without professional activity ☐
- employed ☐
- independent ☐

14. What is your (social) father's highest school-leaving qualification?
- Without school graduation ☐
- secondary schools ☐
- Realschule (10th grade) ☐
- High School ☐

Attachment 1 to: Pelzer A, Sapalidis A, Rabkow N, Pukas L, Günther N, Watzke S. *Does medical school cause depression or do medical students already begin their studies depressed? A longitudinal study over the first semester about depression and influencing factors.* GMS J Med Educ. 2022;39(5):Doc58. DOI: 10.3205/zma001579

15. What is your (social) father's highest professional qualification?

- unskilled ☐
- Vocational training ☐
- Technical / university studies ☐

16. What kind of work is your (social) father currently doing?

- Without professional activity ☐
- employed ☐
- independent ☐

17. How many brothers and sisters do you have?

☐

18. What sibling position are you in?

(Enter a 1 if you are the oldest (or only) child of your parents; a 2 if you are the second oldest etc.) ☐

19. Did you lose a parent through separation or death?

- no ☐
- Separation of parents ☐
- Death of the mother ☐
- Death of the father ☐

20. Who did you grow up with?

- with both birth parents ☐
- grew up with the mother ☐
- grew up with my father ☐
- with mother / father and new partner ☐
- grew up with foster parents ☐

21. What importance did religion play in your upbringing??

- no meaning ☐
- marginal importance ☐
- mediocre importance ☐
- extreme important ☐

22. How important is religion in your life today??

- no meaning ☐
- marginal importance ☐
- mediocre importance ☐
- extreme important ☐

23. Was / is one of your family members receiving treatment for a mental illness?

	**mother**	**father**	**brothers and sisters**	**Grand-parents**
**I do not know**	☐	☐	☐	☐
**none**	☐	☐	☐	☐
**dementia**	☐	☐	☐	☐
**Addiction**	☐	☐	☐	☐
**psychosis**	☐	☐	☐	☐
**Bipolar disorder**	☐	☐	☐	☐
**depression**	☐	☐	☐	☐
**Anxiety disorder**	☐	☐	☐	☐
**eating disorder**	☐	☐	☐	☐
**Personality disorder**	☐	☐	☐	☐

24. Have you been or have you been receiving treatment for a mental illness?

- **none** ☐
- **Disorders in childhood** ☐
- **Addiction** ☐
- **psychosis** ☐
- **Bipolar disorder** ☐
- **depression** ☐
- **Anxiety disorder** ☐
- **eating disorder** ☐
- **Personality disorder** ☐

Questions about your studies

25. Vocational training or studies before studying medicine

- no ☐
- started but not completed ☐
- finished ☐

26 Type of training

- none ☐
- semi-skilled or part skilled worker ☐
- Apprenticeship in craft, production, commercial area ☐
- Technical school training, civil servant training ☐
- University education, university of applied sciences studies ☐

Attachment 1 to: Pelzer A, Sapalidis A, Rabkow N, Pukas L, Günther N, Watzke S. *Does medical school cause depression or do medical students already begin their studies depressed? A longitudinal study over the first semester about depression and influencing factors.* GMS J Med Educ. 2022;39(5):Doc58. DOI: 10.3205/zma001579

27. Activity before starting medical studies

- school education ☐
- vocational training ☐
- other university studies ☐
- professional activities ☐
- voluntary service or year abroad ☐
- no activity ☐

28. Duration of the last activity
in years

☐

29. What were the main reasons for you to choose to study medicine?
(Please select the most important two)

- I found the subject exciting ☐
- I wanted to help people ☐
- I was looking for a job that would give me a lot of respect and recognition ☐
- I was looking for a job with good earning potential and future security ☐
- My parents / family suggested that I study medicine ☐
- I didn't know what else to study ☐

30. Was there a conceivable alternative career or course of study for you?

- Yes ☐
- no ☐

31. How many years did you have to wait for the start of your studies or did you bridge the gap by other means?

☐

32. How did the city where you study rank in your application?

☐

33. Do you enjoy your studies?

- yes, almost always ☐
- most of time ☐
- rarely ☐
- no, almost never ☐

34. Are you sure that you will complete your studies successfully?

- yes, almost always ☐
- most of time ☐
- rarely ☐
- no, almost never ☐

35. From today's perspective, would you decide to study medicine again?

- I am unhappy with my decision ☐
- I would make a different decision today ☐
- The perspective of the medical profession allows me to overcome the hurdles ☐
- I would definitely choose this again ☐

36. Are there things that are currently making it difficult for you to be happy with your decision to study? (Multiple answers possible)

- uncertain future prospects ☐
- competition among students ☐
- little flexibility in structuring studies ☐
- time pressure ☐
- individual courses are very stressful ☐
- financial burden from studying ☐
- Feeling overwhelmed ☐
- loneliness ☐
- Performance pressure ☐

37. How do you currently live?

- alone / own apartment ☐
- Apartment with partner ☐
- in a shared apartment ☐
- Room in student hall ☐
- with the parents ☐

38. If you have moved for your studies, how do you perceive the change of residence?

- positive ☐
- Neutral ☐
- negative ☐
- My place of residence has not changed ☐

39. How do you pay for your studies?
(Multiple answers possible)

- own professional activity / part-time job ☐
- financial support from family ☐
- BAföG ☐
- scholarship ☐
- State social benefits ☐

40. Do you have sufficient financial resources?

- more than enough ☐
- sufficient ☐
- sometimes too little ☐
- often too little ☐
- I am usually under great financial pressure ☐

41. Have you had more or less contact with friends since you started your studies?

- more than before ☐
- Unchanged, a lot ☐
- Unchanged, a little ☐
- less than before ☐

42. Have you had more or less contact with your family since you started your studies?

- more than before ☐
- Unchanged, a lot ☐
- Unchanged, a little ☐
- less than before ☐

43. Are you experiencing sufficient emotional support?

- I get more than enough emotional support ☐
- I get enough emotional support ☐
- Sometimes I don't get enough emotional support ☐
- I often don't get enough emotional support ☐
- I don't get any emotional support ☐

44. How many hours per week do you spend on study-related activities? (Learning, presentation preparation, exam preparation) outside of everyday university life? ☐

45. How many hours per week do you spend on part-time jobs? (0 if you do not have a part-time job) ☐

46. How many hours per week do you spend with your friends, family or your partner? ☐

47. How many hours per week do you spend on your hobbies? ☐

48. How many hours a week do you actively exercise? ☐

49. How many hours a week do you actively play music? ☐

50. How do you rate the time available to you for your studies?

- I always have more than enough time for the requirements of my studies ☐
- I always have enough time for the requirements of my studies ☐
- Sometimes I don't have enough time for the demands of my studies ☐
- I often don't have enough time for the requirements of my studies ☐
- I am usually under time pressure to meet the requirements of the degree ☐

51. How do you rate the time you have available for friends, family and partnership?

- I always have more than enough time for friends, family and partnerships ☐
- I always have enough time for friends, family and partnerships ☐
- Sometimes I don't have enough time for friends, family and partnerships ☐
- I often don't have enough time for friends, family and partnerships ☐
- I am usually under time pressure to maintain friendships, family relationships and partnerships ☐

52. How do you rate the time available to you for your hobbies?

- I always have enough time for the requirements of my studies ☐
- Sometimes I don't have enough time for my hobbies ☐
- I often don't have enough time for my hobbies ☐
- I am usually under time pressure to pursue my hobbies ☐

53. Overall, how satisfied are you with ...

	very satisfied			very dissatisfied
**... your studies**	☐	☐	☐	☐
**... your partnership**	☐	☐	☐	☐
**... your family relationships**	☐	☐	☐	☐
**... your friendships**	☐	☐	☐	☐
**... your opportunity to pursue hobbies**	☐	☐	☐	☐

Attachment 1 to: Pelzer A, Sapalidis A, Rabkow N, Pukas L, Günther N, Watzke S. *Does medical school cause depression or do medical students already begin their studies depressed? A longitudinal study over the first semester about depression and influencing factors.* GMS J Med Educ. 2022;39(5):Doc58. DOI: 10.3205/zma001579

54. Are you satisfied with your diet?

Yes ☐
no ☐

55. Are you happy with your weight?

Yes ☐
no ☐

56. Do you eat regular meals?

Yes ☐
usually ☐
sometimes ☐
no ☐

57. Do you eat a healthy diet?
(For example, do you follow a certain diet?)

Yes ☐
usually ☐
sometimes ☐
no ☐

58. Do you use specific relaxation techniques? (e.g. yoga, PMR, autogenic training, meditation, etc.)

Yes ☐
no ☐

59. How many days a week do you drink at least one alcoholic beverage?

☐

60. How often do you drink more than 6 (Women) or 8 (men) drinks per occasion?
(1 drink = 250 ml beer or 1 glass of wine or 2 cl schnapps)

never ☐
Rare ☐
monthly ☐
weekly ☐
(almost daily ☐

61. In the past year, how often were you unable to (completely) remember an evening after consuming alcohol ("film tear")?

never ☐
Rare ☐
monthly ☐
weekly ☐
(almost daily ☐

62. In the past year, how many times have you been unable to do what you normally would have done on the morning after drinking alcohol?

never ☐
Rare ☐
monthly ☐
weekly ☐
(almost daily ☐

63. How often did someone ask you about your alcohol consumption in the past year? z. B. with the recommendation to reduce consumption?

never ☐
once ☐
several times ☐

64. How many cigarettes do you smoke on average per day?

☐

65. Do you use substances or drugs to improve concentration and performance? (Multiple answers possible)

Beverages containing caffeine ☐
Medication ☐
activating drugs ☐
no ☐

66. Do you use substances or medication to calm down or to be able to sleep?
(Multiple answers possible)

Food (e.g. chocolate) ☐
Medication ☐
sedating drugs ☐
no ☐

67. Which of the following symptoms did you experience during and as a result of your studies? (Multiple answers possible)

Procrastination ☐
Exhaustion ☐
Feeling overwhelmed ☐
Anxiety ☐
Psychosomatic complaints (e.g. gastrointestinal problems, muscle tension, neurodermatitis) ☐
Learning and performance disorders ☐
Identity and self-worth problems ☐
Irritability ☐
Depressive mood ☐

68. Have you spoken to anyone about these symptoms? (Multiple answers possible)

no, I didn't confide in anyone ☐
yes, I confided in someone, namely ...
...my family ☐
...my friends ☐
... my partner ☐
... my fellow students ☐
... my lecturer ☐
... the student advisory service ☐
... a psychotherapist ☐
... a doctor ☐

Attachment 1 to: Pelzer A, Sapalidis A, Rabkow N, Pukas L, Günther N, Watzke S. *Does medical school cause depression or do medical students already begin their studies depressed? A longitudinal study over the first semester about depression and influencing factors.* GMS J Med Educ. 2022;39(5):Doc58. DOI: 10.3205/zma001579
