# Supplementary material for: Does medical school cause depression or do medical students already begin their studies depressed? A longitudinal study over the first semester about depression and influencing factors
Source: GMS J Med Educ. 2022 Aug 4;39(5):Doc58. doi: 10.3205/zma001579 (PMC9733480; doi:10.3205/zma001579)
Supplement: Sociodemography, Risk & Resilience. Self-description questionnaire (T1) [file JME-39-58-s-002.pdf]

SOCIODEMOGRAPHIC QUESTIONS
STRESS & RESILIENCE FACTORS

Self-description questionnaire (T1)

In order to be able to link the data of today's survey with those of previous surveys without anyone being able to find out who answered the questions, we again ask you to provide your personal code:

- First and last letter of your mother's first name ___ ___
- Day of your mother's birth (two digits) ___ ___
- First and second letter of your mother's birthplace ___ ___

PNUM: (is assigned by the study team) Date:

Please answer each of the following items by checking the applicable alternative or writing in the applicable value.

1. biological gender
- female ☐
- male ☐

2. civil status
- single ☐
- married ☐
- separated or divorced ☐
- widowed ☐

3. age in years ☐

4. size in cm ☐

5. weight in kg ☐

6. Relationship regardless of marital status
- no partner or relationship shorter than 3 months / living without a partner ☐
- married and living together ☐
- not married, but living together ☐
- partnership without living together (> 3 months) ☐

7. Duration of the relationship in months (0 if no partnership) ☐

8. Number of biological children ☐

9. Are you currently receiving treatment for a mental illness?
- none ☐
- depression ☐
- bipolar disorder ☐
- psychosis ☐
- anxiety disorder ☐
- addicition ☐
- eating disorder ☐
- personality disorder ☐

10. Do you enjoy your studies?
- yes, almost always ☐
- most of time ☐
- rarely ☐
- no, almost never ☐

11. Are you sure that you will complete your studies successfully?
- yes, almost always ☐
- most of the time ☐
- rarely ☐
- no, almost never ☐

12. From today's perspective, would you decide to study medicine again?
- I am unhappy with my decision ☐
- I would make a different decision today ☐
- The perspective of the medical profession allows me to overcome the hurdles ☐
- I would definitely choose this again ☐

13. Are there things that are currently making it difficult for you to be happy with your decision to study? (Multiple answers possible)
- uncertain future prospects ☐
- competition among students ☐
- little flexibility in structuring studies ☐
- time pressure ☐
- individual burden from studying ☐
- financial burden from studying ☐
- feeling overwhelmed ☐
- loneliness ☐
- performance pressure ☐

Attachment 2 to: Pelzer A, Sapalidis A, Rabkow N, Pukas L, Günther N, Watzke S. *Does medical school cause depression or do medical students already begin their studies depressed? A longitudinal study over the first semester about depression and influencing factors.* GMS J Med Educ. 2022;39(5):Doc58. DOI: 10.3205/zma001579

14. How do you currently live?

- alone / own apartment ☐
- apartment with my partner ☐
- in a shared apartment ☐
- room in student hall ☐
- with the parents ☐

15. If you have moved for your studies, how do you perceive the change of residence?

- positive ☐
- neutral ☐
- negative ☐
- My place of residence has not changed ☐

16. How do you pay for your studies?
(Multiple answers possible)

- own professional activity / part-time job ☐
- financial support from family ☐
- BAföG ☐
- scholarship ☐
- State social benefits ☐

17. Do you have sufficient financial resources?

- more than enough ☐
- sufficient ☐
- sometimes too little ☐
- often too little ☐
- I am usually under great financial pressure ☐

18. Have you had more or less contact with friends since you started your studies?

- more than before ☐
- unchanged, a lot ☐
- unchanged, a little ☐
- less than before ☐

19. Have you had more or less contact with your family since you started your studies?

- more than before ☐
- unchanged, a lot ☐
- unchanged, a little ☐
- less than before ☐

20. Are you experiencing sufficient emotional support?

- I get more than enough emotional support ☐
- I get enough emotional support ☐
- Sometimes I don't get enough emotional support ☐
- I often don't get enough emotional support ☐
- I don't get any emotional support ☐

21. How many hours per week do you spend on part-time jobs? (0 if you do not have a part-time job)

☐

22. How many hours per week do you spend on study-related activities outside of everyday university life? (Learning, presentation preparation, exam preparation)

☐

23. How many hours per week do you spend with your friends, family or your partner?

☐

24. How many hours per week do you spend on your hobbies?

☐

25. How many hours a week do you actively exercise?

☐

26. How many hours a week do you actively play music?

☐

27. How do you rate the time available to you for your studies?

- I always have more than enough time for the requirements of my studies ☐
- I always have enough time for the requirements of my studies ☐
- Sometimes I don't have enough time for the demands of my studies ☐
- I often don't have enough time for the requirements of my studies ☐
- I am usually under time pressure to meet the requirements of the degree ☐

28. How do you rate the time you have available for friends, family and partnership?

- I always have more than enough time for friends, family and partnerships ☐
- I always have enough time for friends, family and partnerships ☐
- Sometimes i don't have enough time for friends, family and partnerships ☐
- I often don't have enough time for friends, family and partnerships ☐
- I am usually under time pressure to maintain friendships, family relationships and partnerships ☐

29. How do you rate the time available to you for your hobbies?

- I always have enough time for the requirements of my studies ☐
- Sometimes I don't have enough time for my hobbies ☐
- I often don't have enough time for my hobbies ☐
- I am usually under time pressure to pursue my hobbies ☐

30. Overall, how satisfied are you with…

	very satisfied			very dissatisfied
**… your studies**	☐	☐	☐	☐
**… your partnership**	☐	☐	☐	☐
**… your family relationships**	☐	☐	☐	☐
**… your friendships**	☐	☐	☐	☐
**… your opportunity to pursue hobbies**	☐	☐	☐	☐

31. Are you satisfied with your diet?

- yes ☐
- no ☐

32. Are you happy with your weight?

- yes ☐
- no ☐

33. Do you eat regular meals?

- yes ☐
- usually ☐
- sometimes ☐
- no ☐

34. Do you eat a healthy diet?
(For example, do you follow a certain diet?)

- yes ☐
- usually ☐
- sometimes ☐
- no ☐

35. Do you use specific relaxation techniques? (e.g. yoga, PMR, autogenic training, meditation, etc.)

- yes ☐
- no ☐

36. How many days a week do you drink at least one alcoholic beverage? ☐

37. How often do you drink more than 6 (women) **or 8** (men) **drinks per occasion?** (1 drink = 250 ml beer or 1 glass of wine or 2 cl schnapps)

- never ☐
- rare ☐
- monthly ☐
- weekly ☐
- (almost) daily ☐

38. In the past year, how often were you unable to (completely) remember an evening after consuming alcohol ("film tear")?

- never ☐
- rare ☐
- monthly ☐
- weekly ☐
- (almost) daily ☐

39. In the past year, how many times have you been unable to do what you normally would have done on the morning after drinking alcohol?

- never ☐
- rare ☐
- monthly ☐
- weekly ☐
- (almost) daily ☐

40. How often did anyone ask you about your alcohol consumption in the past year? E.g. with the recommendation to reduce consumption?

- never ☐
- once ☐
- several times ☐

41. How many cigarettes do you smoke on average per day? ☐

Attachment 2 to: Pelzer A, Sapalidis A, Rabkow N, Pukas L, Günther N, Watzke S. *Does medical school cause depression or do medical students already begin their studies depressed? A longitudinal study over the first semester about depression and influencing factors.* GMS J Med Educ. 2022;39(5):Doc58. DOI: 10.3205/zma001579

42. Do you use substances or drugs to improve concentration and performance?
(Multiple answers possible)

beverages containing caffeine	☐
medication	☐
activating drugs	☐
no	☐

43. Do you use substances or medication to calm down or to be able to sleep?
(Multiple answers possible)

Food (e.g. chocolate)	☐
medication	☐
sedating drugs	☐
no	☐

44. Which of the following medications have you taken in the past 12 months?
Please tick everything that applies to you!

painkillers	☐	appetite suppressants	☐
sleeping pills	☐	antidepressants	☐
tranquilizer	☐	neuroleptics	☐
stimulants	☐	anabolics	☐

45. Which of the following medications have you taken in the past 12 months?
Please tick everything that applies to you!

	How often taken in the last 30 days?					Was the intake prescribed by the doctor ?	
	Not taken	Less frequently than once a week	several times a week	daily		yes	no
painkillers	☐	☐	☐	☐	⇨	☐	☐
Sleeping pills	☐	☐	☐	☐	⇨	☐	☐
tranquilizer	☐	☐	☐	☐	⇨	☐	☐
stimulants	☐	☐	☐	☐	⇨	☐	☐
Appetite suppressants	☐	☐	☐	☐	⇨	☐	☐
antidepressants	☐	☐	☐	☐	⇨	☐	☐
neuroleptics	☐	☐	☐	☐	⇨	☐	☐
anabolics	☐	☐	☐	☐	⇨	☐	☐

46. Which of the following symptoms did you experience during and as a result of your studies?
(Multiple answers possible)

Procrastination	☐
Exhaustion	☐
Feeling overwhelmed	☐
Anxiety	☐
Psychosomatic complaints (e.g. gastrointestinal problems, muscle tension, neurodermatitis)	☐
Learning and performance disorders	☐
Identity and self-worth problems	☐
Irritability	☐
Depressive mood	☐

47. Have you spoken to anyone about these symptoms? (Multiple answers possible)

No, i didn’t confide in anyone	☐
Yes, I confided in someone, namely	
…my family	☐
…my friends	☐
…my partner	☐
…my fellow students	☐
…my lecturer	☐
…the student advisory service	☐
…a psychotherapist	☐
…a doctor	☐
